# NLRP3+ macrophages aggravate inflammatory cystitis in diabetes

**DOI:** 10.3389/fimmu.2022.1057746

**Published:** 2022-11-03

**Authors:** Yubing Peng, Yan Gao

**Affiliations:** ^1^ Department of Urology, RenJi Hospital Affiliated to Shanghai Jiao Tong University School of Medicine, Shanghai, China; ^2^ Department of Infectious Diseases, HuaShan Hospital, Fudan University, Shanghai, China

**Keywords:** inflammatory cystitis, diabetes, macrophages, inflammation, NOD-, LRR-and PYD domains-containing protein 3 (NLRP3)

## Abstract

Inflammatory macrophages play a pivotal role in the progression of inflammatory cystitis. Formation of NOD-, LRR- and PYD domains-containing protein 3 (NLRP3) inflammasome triggers the activation of caspase-1/IL-1β signaling cascades to mediate inflammatory response. However, it is not known whether NLRP3 activation in macrophages during cystitis may differ in normal or diabetic setting as well as the importance of it. In this study, we found that NLRP3 levels significantly increased in bladder macrophages in diabetic mice that underwent cystitis. Moreover, bladder macrophages from diabetic mice appeared to have increased their potential of growth, migration and phagocytosis. Furthermore, specific depletion of NLRP3 in macrophages alleviated the severity of cystitis in diabetic mice, but not in non-diabetic mice. Together, our data suggest that NLRP3 depletion in macrophages may be a promising strategy for treating diabetic cystitis.

## Introduction

Interstitial cystitis (IC) and bladder pain syndrome (BPS) are clinical syndromes manifested with frequent urgency, urination, accompanied with discomfort in the suprapubic bladder area (1). The majority of IC/PBS patients are women, with a male-to-female ratio of about 1:10 (2). In 2011, Berry et al. found that the prevalence of IC/BPS was about 2.7% to 6.5% based on large epidemiological data of the adult female population in the United States (3). IC/BPS seriously affects the quality of life of patients. Chronic pain, avoidance of sex, and poor sleep quality directly affect work and life (4). The etiology of IC/PBS is unknown, although interstitial cystitis has been regarded as a chronic inflammatory disorder.

To improve our current understanding of the pathophysiology of IC/BPS, many animal models have been used (5). So far, cyclophosphamide (CYP)-induced urinary bladder disorder is one of the best experimental models for IC/BPS (6). Of note, CYP-induced cystitis model causes a local inflammation associated with most symptoms detected in IC/BPS in human patients (7). Therefore, the CYP-induced cystitis model appears to be widely used in rodents to study the mechanisms underlying the pathological changes in cystitis (8).

Diabetic patients are prone to getting IC/BPS, and they are often suffered from other bladder issues such as frequent urinary tract infection (UTI) and neurogenic bladder, for which the patients lose the control of their bladder due to issues from the brain, spinal cord, or nerves (9). However, the mechanism remains largely unknown. Very recently, it is recognized that inflammatory macrophages play a pivotal role in the pathological progression of cystitis (10). This finding brought a breakthrough for understanding the effects of diabetes on IC/BPS, since diabetes is well-known metabolic disease associated with dysfunction in immunological regulation of the body (11).

The display of macrophage phenotype to exhibit either a pro-inflammatory phenotype, which is called “M1”, or an anti-inflammatory phenotype, which is called “M2”, is known as “polarization of a macrophage” (12). The newly generated macrophages with any polarization are naïve macrophages (M0) (13). Interleukin 1β (IL-1β) is a key cytokine that regulate inflammation (14). The effects of IL-1β on inflammation are complex and context-sensitive. Inflammasomes are known regulators for IL-1β activation (15). The most important inflammasome is NOD-, LRR- and PYD domains-containing protein 3 (NLRP3), which promotes the dimerization of caspase-1 to catalyze the pro-IL-1β into IL-1β to be active (16). However, it is not known whether NLRP3 activation in macrophages during IC may differ in normal or a diabetic setting as well as the importance of it. This question was hence addressed in the current study.

## Materials and methods

### Ethic approval

All the experiments including animal work have been approved by and performed according to the guideline by institutional Animal Research Ethics Council from Shanghai Jiao Tong University School of Medicine. This study did not involve human specimens.

### Animals

Mice with macrophage-depletion of NLRP3 were generated using Lysosome2 (Lys2)-Cre (Strain #:018956; The Jackson Laboratory, Bar Harbor, ME, USA) and NLRP3(fx/fx) (Strain #:017970; The Jackson Laboratory). The NLRP3(fx/fx) mice without Lys-Cre were used as controls. C57/Bl6 mice were used as wildtype mice. In all experiments, both male and female mice at age of 10 weeks were used and evenly distributed in the experimental groups. After an overnight fasting that exceeds 16 hours (D0), one intraperitoneal (i.p) injection of 110 mg/kg streptozotocin (STZ) in 150µl normal saline was performed in mice to induce diabetes. An equal volume of normal saline was given i.p. to control mice. An overnight fasting was required for both fasting blood glucose measurement and intraperitoneal glucose tolerance testing (IPGTT). For IPGTT, 2mg glucose was challenged to the mice, after which glucose levels were determined at different time points. Cystitis was induced by i.p. injection of CYP (70 mg/kg, Fisher-Scientific, Pittsburgh, PA, USA) 4 times on D6, 8, 10 and 12. Control animals received saline of equal volume. Von Frey test was done at D13 according to published protocols. Briefly, mice were put on a raised wire mesh floor to allow at least 30 minutes’ acclimatization before starting the test. Nociceptive response was evaluated after the mouse abdomen close to urinary bladder were challenged with several von Frey filaments of increasing forces. The scoring of nociceptive response was as 0 for no response, as 1 for detection of some reaction, as 2 for detection of mouse reaction and position changing, and as 3 for detection of licking or vocalizing of stimulated area in addition to those for 2. Nociceptive score was presented as a relative value. Beta-cell mass was assessed at sacrifice (D16) by calculating the product of the percentage of the area positive for insulin staining (Abcam, San Jose, CA, USA) to the total pancreatic area with the pancreatic weight. For the first experiment, 4 groups of C57/Bl6 mice of 6 of each were used. Group 1: mice received i.p. saline (control for STZ) at D0 and i.p. saline (control for CYP) at D6, 8, 10 and 12 (saline+saline); Group 2: mice received i.p. STZ at D0 and i.p. saline (control for CYP) at D6, 8, 10 and 12 (STZ+saline); Group 3: mice received i.p. saline (control for STZ) at D0 and i.p. CYP at D6, 8, 10 and 12 (saline+CYP); Group 4: mice received i.p. STZ at D0 and i.p. CYP at D6, 8, 10 and 12 (STZ+CYP). For the second experiment, 4 groups of Lys2-Cre; NLRP3(fx/fx) or NLRP3(fx/fx) mice of 6 of each were used. Group 1: NLRP3(fx/fx) mice received i.p. saline (control for STZ) at D0 and i.p. CYP at D6, 8, 10 and 12 (saline+CYP/N); Group 2: Lys2-Cre; NLRP3(fx/fx) mice received i.p. saline (control for STZ) at D0 and i.p. CYP at D6, 8, 10 and 12 (saline+CYP/LN); Group 3: NLRP3(fx/fx) mice received i.p. STZ at D0 and i.p. CYP at D6, 8, 10 and 12 (STZ+CYP/N); Group 4: Lys2-Cre; NLRP3(fx/fx) mice received i.p. STZ at D0 and i.p. CYP at D6, 8, 10 and 12 (STZ+CYP/LN).

### Assessment of bladder weight, edema and vascular permeability inflammation

At sacrifice (D16), mouse bladders were immediately collected and measured for bladder weight, edema and vascular permeability Inflammation. Edema score was obtained as 0 for no edema, 1 for mild edema, 2 for moderate edema and 3 for severe edema. Vesical vascular permeability was evaluated by the Evans blue extravasation technique on the sectioned bladder slides. Briefly, Evans blue (20 mg/kg) was injected from tail vein 30 minutes before post-perfusion sacrifice and then measured by tissue absorbance at 620 nm.

### Flow cytometry

For flow cytometry analysis, mouse bladder was dissected out and dissociated into a single cell population with 40 minutes’ treatment with 0.25% Trypsin (Invitrogen) at 37°C. The cells were then labeled with PE-cy5-conjugated F4/80 and FITC-conjugated CD163 antibodies (Becton-Dickinson Biosciences, Shanghai, China). Flow cytometry data were analyzed and presented by FlowJo software (Flowjo LLC, Ashland, OR, USA).

### ELISA

Total protein was extracted from FACS-isolated cells to be used for ELISA analysis with specific kits for mouse NLRP3 (ab279417; Abcam, Cambridge, MA, USA), nitric oxide synthase (iNOS, ab253219; Abcam), tumor necrosis factor alpha (TNFα, ab208348; Abcam), interferon gamma (IFNγ, ab282874; Abcam), CD163 (ab272204; Abcam) and proliferating cell nuclear antigen (PCNA, ab196270; Abcam).

### Cell proliferation and migration assay

Cell growth was determined by viable cell number with a Cell Counting Kit-8 assay (CCK-8, Sigma-Aldrich). Cell migration was determined by a transwell cell migration assay, as reported (17). Briefly, cells were plated at 5X10^3^ cells/cm^2^ in the corresponding culture vessel using RPMI1640 culture medium (Invitrogen), after which the cell culture inserts were put into the wells of the 24-well plate with 750μL medium with 10% FBS to the outer compartment. The transmigration was performed in a humidified incubator (37°C, 5% CO_2_) for 4 hours, followed by cell fixation and staining of the migrated cells with Crystal Violet (V5265, Sigma-Aldrich) for 20 minutes at room temperature.

### Analysis of phagocytosis

Phagocytosis was assessed based on 30 minutes’ zymosan intake by macrophages using a zymosan-based phagocytotic kit (ab211156, Abcam), or based on intake of GFP+ bacteria with a flow cytometry-based method as described (18).

### Statistics and bioinformatics

A GEO database GSE183698 was used to analyzed gene profile from mouse macrophages with or without NLRP3 depletion. R software was used to analyze differentiated genes, while String online tools were used to analyze gene interaction network. All data were statistically analyzed with GraphPad Prism 7 (GraphPad, Chicago, IL, USA). One-way ANOVA method was applied for determining the significance (p<0.05) or no significance (ns, p<0.05). Individual values, the mean and standard deviation (SD) were all shown in the figures.

## Results

### Diabetes aggravates CYP-induced cystitis

**Figure 1:**
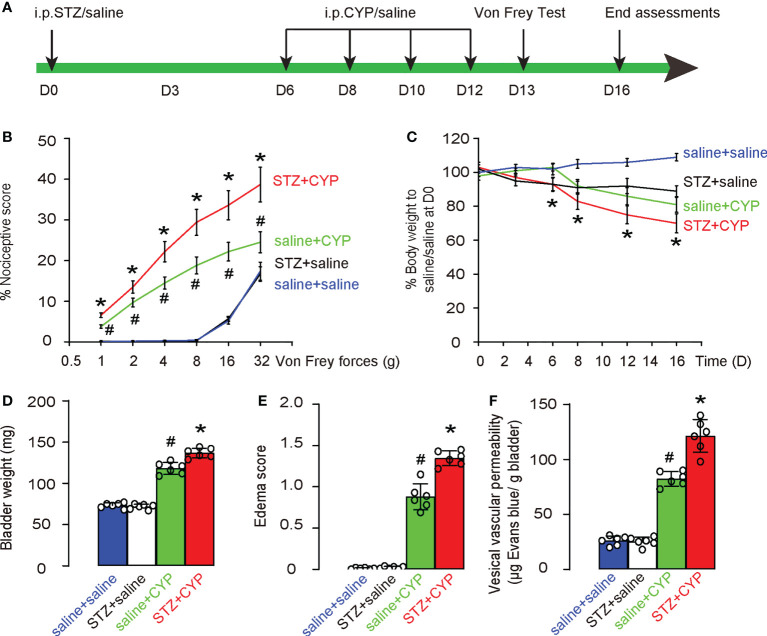
Diabetes aggravates CYP-induced cystitis. **(A)** Schematic of experiment: STZ was used at D0 to develop diabetes in mice. After 6 days when diabetes was confirmed, experimental cystitis was induced by 4 i.p. injection of CYP on D6, 8, 10 and 12. Von Frey test was done at D13, and mice were sacrificed at D16 for end assessments. **(B-F)** Four groups of mice were used. Group 1: mice received i.p. saline (control for STZ) at D0 and i.p. saline (control for CYP) at D6, 8, 10 and 12 (saline+saline); Group 2: mice received i.p. STZ at D0 and i.p. saline (control for CYP) at D6, 8, 10 and 12 (STZ+saline); Group 3: mice received i.p. saline (control for STZ) at D0 and i.p. CYP at D6, 8, 10 and 12 (saline+CYP); Group 4: mice received i.p. STZ at D0 and i.p. CYP at D6, 8, 10 and 12 (STZ+CYP). **(B)** Nociceptive score of the mice at Von Frey test at D13. **(C)** Changes in body weight. **(D)** Bladder weight at D16. **(E)** Edema score of the bladder at D16. **(F)** Vesical vascular permeability in bladder at D16. *p<0.05 (STZ+CYP vs saline+CYP). #p<0.05 (saline+CYP vs saline+saline or STZ+saline).

In order to study the molecular mechanisms underlying the diabetic cystitis, we applied a most commonly used rodent model for cystitis. STZ was used at D0 to develop diabetes in mice. After 6 days when diabetes was confirmed, experimental cystitis was induced by 4 i.p. injection of CYP on D6, 8, 10 and 12. Von Frey test was done at D13, and mice were sacrificed at D16 for end assessments (**Figure 1A**). Four groups of mice were used. Group 1: mice received i.p. saline (control for STZ) at D0 and i.p. saline (control for CYP) at D6, 8, 10 and 12 (saline+saline); Group 2: mice received i.p. STZ at D0 and i.p. saline (control for CYP) at D6, 8, 10 and 12 (STZ+saline); Group 3: mice received i.p. saline (control for STZ) at D0 and i.p. CYP at D6, 8, 10 and 12 (saline+CYP); Group 4: mice received i.p. STZ at D0 and i.p. CYP at D6, 8, 10 and 12 (STZ+CYP). In saline-treated group (control of CYP), diabetes did not alter the nociceptive score of the mice at Von Frey test, while in CYP-treated group, diabetes significantly worsened the nociceptive score of the mice at Von Frey test (**Figure 1B**). Moreover, in saline-treated group (control of CYP), diabetes did not cause the changes in body weight, while in CYP-treated group, diabetes caused significantly more loss of the body weight (**Figure 1C**). At sacrifice, we found that in saline-treated group (control of CYP), diabetes did not alter the bladder weight (**Figure 1D**), edema score of the bladder (**Figure 1E**) or the vesical vascular permeability in bladder (**Figure 1F**), while in CYP-treated group, diabetes significantly increased bladder weight (**Figure 1D**), edema score of the bladder (**Figure 1E**) or the vesical vascular permeability in bladder (**Figure 1F**). Together, these data suggest that diabetes aggravates CYP-induced cystitis.

### CYP-induced cystitis does not affect diabetic status in mice

**Figure 2:**
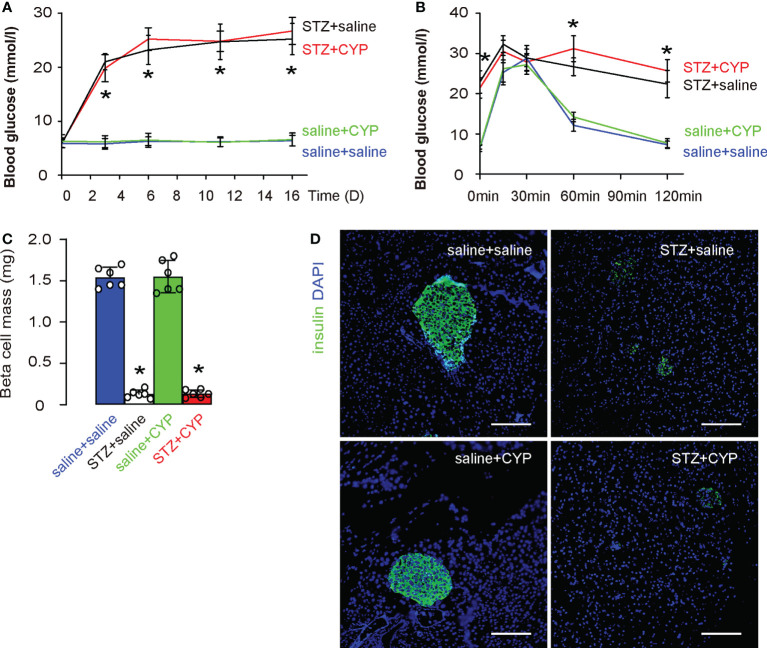
CYP-induced cystitis does not affect diabetic status in mice. **(A)** Fasting blood glucose. **(B)** IPGTT at D16. **(C)** Beta cell mass at D16. **(D)** Representative insulin staining in mouse pancreas. *p<0.05 (STZ+CYP vs saline+CYP or STZ+saline vs saline+saline). Scale bars are 100µm.

To exclude a possibility that the CYP treatment may affect the diabetic status in mice, we monitored the changes in fasting blood glucose, and examined the end point glucose response of mice as well as beta cell mass. We found that CYP-treatment did not alter fasting blood glucose (**Figure 2A**), the end point glucose response of mice (**Figure 2B**), as well as beta cell mass (**Figures 2C, D**). These data suggest that CYP-induced cystitis does not affect diabetic status in mice.

### NLRP3 significantly increases in bladder macrophages in diabetic mice that undergo cystitis

**Figure 3:**
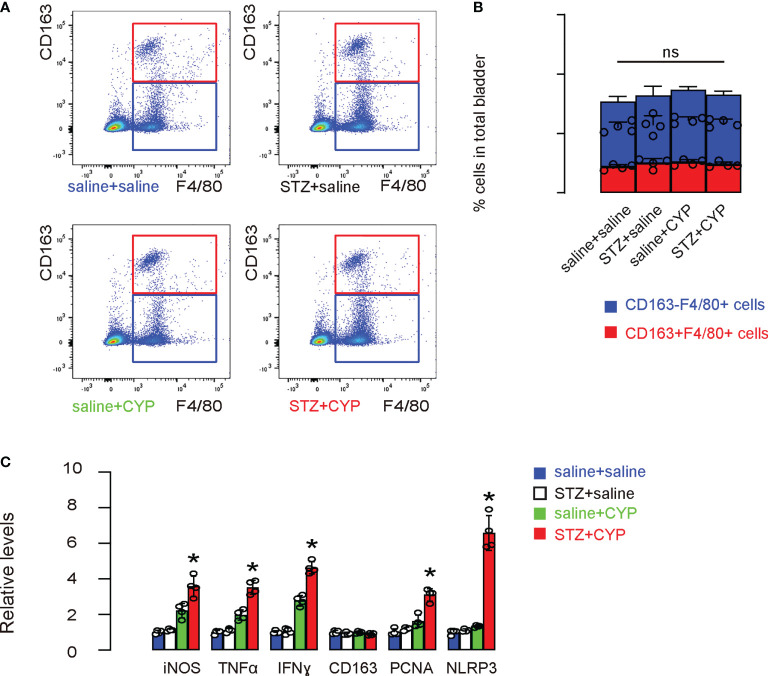
NLRP3 significantly increases in bladder macrophages in diabetic mice that undergo cystitis. **(A, B)** Mouse bladders were sorted for bladder macrophages based on positivity for F4/80, and further for M1 (CD163-) and M2 (CD163+) subtypes among all F4/80+ macrophages, shown by representative flow charts **(A)** and by quantification **(B)**. **(C)** ELISA for some critical factors for macrophage phenotype and functionality. *p<0.05. ns: no significance.

Next, we aimed to figure out whether bladder macrophages could be affected by diabetes and then play a role in the aggravation of the cystitis. Thus, we digested mouse bladders and analyzed and sorted bladder macrophages based on positivity for F4/80, and further distinguished M1 (CD163-) and M2 (CD163+) subtypes among all F4/80+ macrophages. We did not detect significant changes in the total macrophage numbers or M1/M2 distributions in all 4 groups (**Figures 3A, B**). However, when the sorted bladder macrophages were analyzed for some critical genes for macrophage phenotype and functionality, we found that diabetic status of the mice caused increases in iNOS, TNFα and IFNγ, 3 factors associated with proinflammatory function and phagocytosis of macrophage, increases in PCNA, a cell proliferation marker, and a dramatic increase in NLRP3, without changing CD163, a M2 macrophage marker (**Figures 3C**). These data suggest that although diabetes did not cause a major shift of macrophage polarity, it appeared to significantly alter the function of macrophages.

### NLRP3 depletion in macrophages alters genes associated with macrophage phenotype

**Figure 4:**
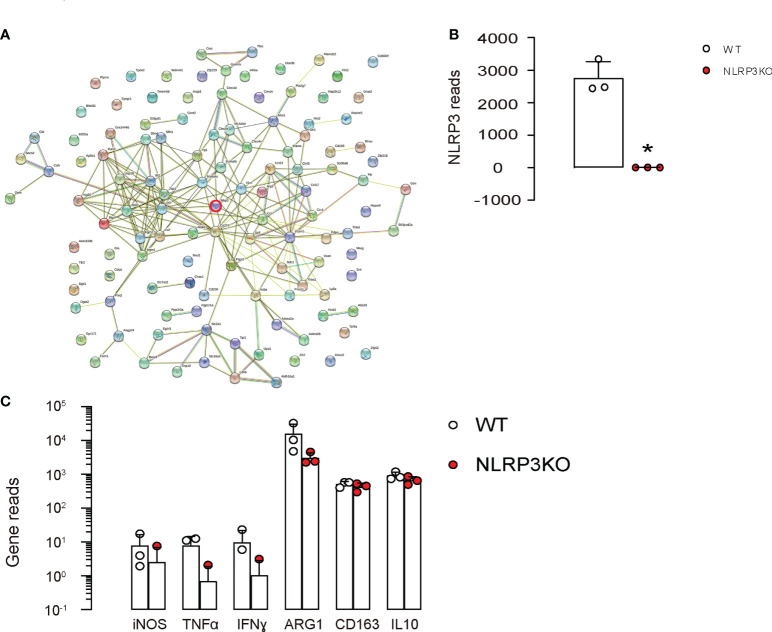
NLRP3 depletion in macrophages alters genes associated with macrophage phenotype. **(A-C)** A GEO database GSE183698 was used to analyzed gene profile from mouse macrophages with or without NLRP3 depletion (NLRP3KO, WT). **(A)** Genes associated with fine determination of macrophage phenotype analyzed by String online tool. **(B, C)** Gene reads from NLRP3 and some key factors associated with macrophage phenotype and functionality. *p<0.05.

In order to determine whether NLRP3 may be responsible for the phenotypic changes in macrophages under diabetes, a GEO database GSE183698 was used to analyzed gene profile from mouse macrophages with or without NLRP3 depletion (NLRP3KO, WT). In this database, in all significantly upregulated and significantly downregulated genes (p<0.05; logFC>1 or logFC<-1), there were 120 genes associated with fine determination of macrophage phenotype (**Figure 4A**) analyzed by String online tool. However, although NLRP3 depletion (**Figure 4B**) resulted in alteration in 120 genes associated with macrophage polarization, none of the key M1/M2 markers [iNOS, TNFα, IFNγ, arginase 1 (ARG1), CD163 and IL-10] were significantly altered (**Figure 4C**), suggesting that depletion of NLRP3 may result in some delicate changes in the characteristics of macrophages, but not result in a major M1/M2 shift.

### Diabetes increases growth and migration potential of macrophages from inflammatory bladder

**Figure 5:**
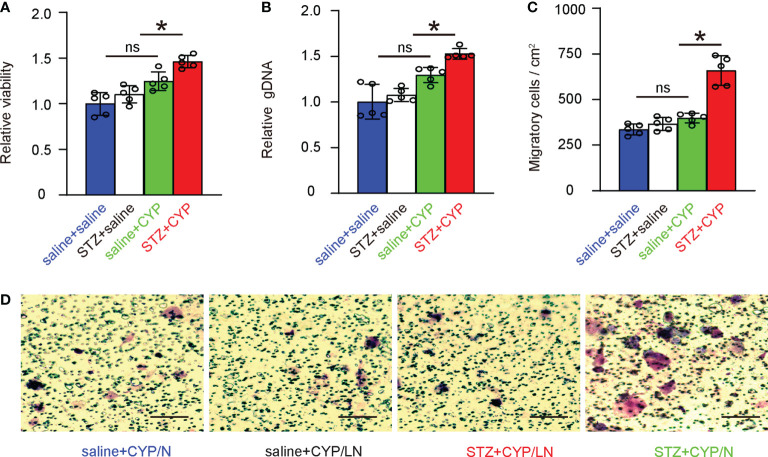
Diabetes increases growth and migration potential of macrophages from inflammatory bladder. **(A, B)** Bladder macrophages from 4 groups of the mice were analyzed for cell growth in a CCK-8 assay **(A)** and in assay to determine DNA **(B)**. **(C, D)** Bladder macrophages from 4 groups of the mice were analyzed for migration, shown by quantification **(C)** and by representative images **(D)**. *p<0.05. ns: no significance. Scale bars are 100µm.

Next, bladder macrophages from 4 groups of the mice were subjected to analysis on cell growth and migration. We found that diabetes increased the growth of bladder macrophages from CYP-treated mice, but not in bladder macrophages from mice without cystitis (**Figures 5A, B**). Similarly, diabetes increased the migration of bladder macrophages from CYP-treated mice, but not in bladder macrophages from mice without cystitis (**Figures 5C, D**). These data were consistent with analysis on the key factors in macrophages (**Figures 3C**), suggesting that diabetes increases growth and migration potential of macrophages from inflammatory bladder.

### Diabetes increases phagocytosis of macrophages from inflammatory bladder

**Figure 6:**
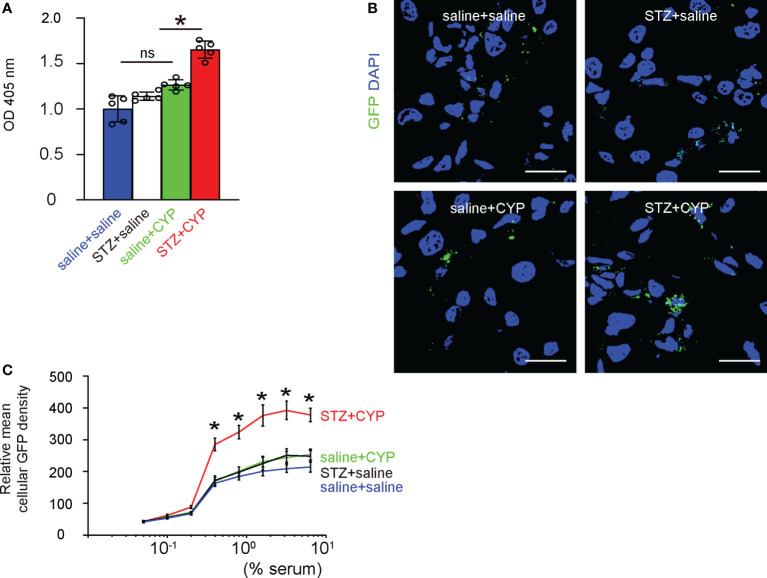
Diabetes increases phagocytosis of macrophages from inflammatory bladder. **(A)** A phagocytosis assay that analyzes 30 minutes’ zymosan intake. **(B, C)** Analysis of the intake of GFP+ bacteria, shown by representative images **(B)**, by the mean cellular GFP density of macrophages **(C)**. *p<0.05. Scale bars are 15µm. ns, no significance.

Phagocytosis is a major function of macrophages and is related to levels of inflammatory responses and resolution. We thus analyzed the phagocytosis of bladder macrophages from 4 groups of the mice. First, we used a phagocytosis assay that analyzes 30 minutes’ zymosan intake, which showed that diabetes significantly increased the phagocytosis of bladder macrophages from CYP-treated mice, but not in bladder macrophages from mice without cystitis (**Figures 6A, B**). Next, we analyzed the intake of GFP+ bacteria by the bladder macrophages from 4 groups, showing that diabetes significantly increased the mean cellular GFP density from CYP-treated mice, but not in bladder macrophages from mice without cystitis (**Figure 6C**), suggesting that diabetes increases phagocytosis of macrophages from inflammatory bladder, also consistent with analysis on the key factors in macrophages (**Figure 3C**).

### Specific depletion of NLRP3 in macrophages alleviates the severity of cystitis in diabetic mice

**Figure 7:**
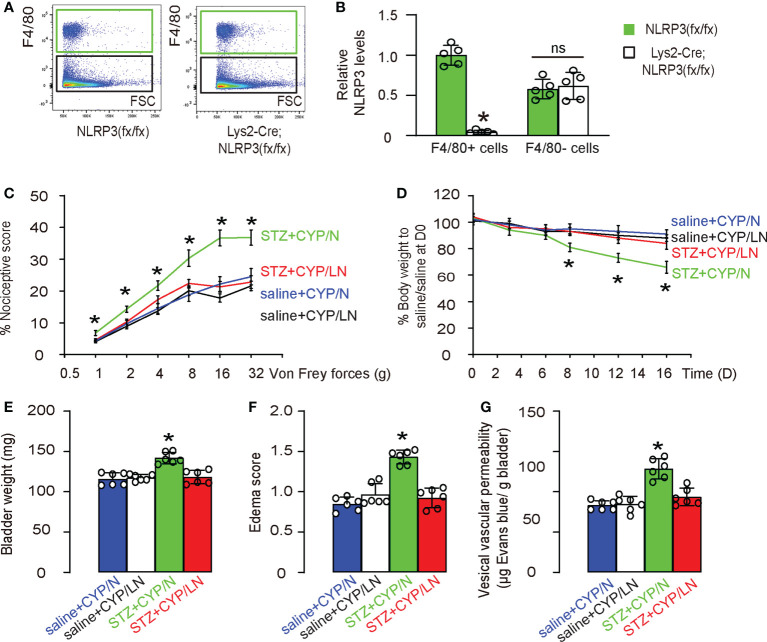
Specific depletion of NLRP3 in macrophages alleviates the severity of cystitis in diabetic mice. We generated mice with macrophage-depletion of NLRP3, Lys2-Cre; NLRP3(fx/fx) and control NLRP3(fx/fx). **(A)** Flow cytometry showing isolation of macrophages from the bladders of these mice. **(B)** ELISA for NLRP3 in macrophages nor non-macrophages from the mouse bladder. **(C)** STZ was used at D0 to develop diabetes in mice. After 6 days when diabetes was confirmed, experimental cystitis was induced by 4 i.p. injection of CYP on D6, 8, 10 and 12. Von Frey test was done at D13, and mice were sacrificed at D16 for end assessments. Group 1: NLRP3(fx/fx) mice received i.p. saline (control for STZ) at D0 and i.p. CYP at D6, 8, 10 and 12 (saline+CYP/N); Group 2: Lys2-Cre; NLRP3(fx/fx) mice received i.p. saline (control for STZ) at D0 and i.p. CYP at D6, 8, 10 and 12 (saline+CYP/LN); Group 3: NLRP3(fx/fx) mice received i.p. STZ at D0 and i.p. CYP at D6, 8, 10 and 12 (STZ+CYP/N); Group 4: Lys2-Cre; NLRP3(fx/fx) mice received i.p. STZ at D0 and i.p. CYP at D6, 8, 10 and 12 (STZ+CYP/LN). **(C)** Nociceptive score of the mice at Von Frey test at D13. **(D)** Changes in body weight. **(E)** Bladder weight at D16. **(F)** Edema score of the bladder at D16. **(G)** Vesical vascular permeability in bladder at D16. *p<0.05. ns: no significance.

Finally, we wanted to understand whether the over-activation of NLRP3 in macrophages during diabetic cystitis may be important to the increased severity of the disease. For this reason, we generated mice with macrophage-depletion of NLRP3, Lys2-Cre; NLRP3(fx/fx) and control NLRP3(fx/fx). Macrophages were isolated with the bladders of these mice (**Figure 7A**) and confirmed for specific depletion of NLRP3 in macrophages from Lys2-Cre; NLRP3(fx/fx) mice (**Figure 7B**). Next, we applied the CYP model to these mice. Again, STZ was used at D0 to develop diabetes in mice. After 6 days when diabetes was confirmed, experimental cystitis was induced by 4 i.p. injection of CYP on D6, 8, 10 and 12. Von Frey test was done at D13, and mice were sacrificed at D16 for end assessments. Group 1: NLRP3(fx/fx) mice received i.p. saline (control for STZ) at D0 and i.p. CYP at D6, 8, 10 and 12 (saline+CYP/N); Group 2: Lys2-Cre; NLRP3(fx/fx) mice received i.p. saline (control for STZ) at D0 and i.p. CYP at D6, 8, 10 and 12 (saline+CYP/LN); Group 3: NLRP3(fx/fx) mice received i.p. STZ at D0 and i.p. CYP at D6, 8, 10 and 12 (STZ+CYP/N); Group 4: Lys2-Cre; NLRP3(fx/fx) mice received i.p. STZ at D0 and i.p. CYP at D6, 8, 10 and 12 (STZ+CYP/LN). We found that macrophage-depletion of NLRP3 did not alter the nociceptive score of the mice at Von Frey test in non-diabetic mice, but significantly increased the nociceptive score of the mice at Von Frey test in non-diabetic mice (**Figure 7C**). Moreover, macrophage-depletion of NLRP3 did not cause the changes in body weight of non-diabetic mice, but significantly decreased the body weight of diabetic mice (**Figure 7D**). At sacrifice, we found that macrophage-depletion of NLRP3 did not alter the bladder weight (**Figure 7E**), edema score of the bladder (**Figure 7F**) or the vesical vascular permeability in bladder (**Figure 7G**) of non-diabetic mice, but significantly increased bladder weight (**Figure 7E**), edema score of the bladder (**Figure 7F**) or the vesical vascular permeability in bladder (**Figure 7G**) of diabetic mice. Together, these data suggest that specific depletion of NLRP3 in macrophages alleviates the severity of cystitis in diabetic mice.

## Discussion

Diabetes increases the incidence and severity of IC/BPS (19). The recognition of the importance of local inflammation to the development of cystitis highlights the importance of studies of the major player for innate immunity, bladder macrophages (20). A recent study has demonstrated a 6-fold increase in bladder monocytes/macrophages in IC/BPS patients compared to healthy controls (21). However, the exact molecular signaling that underlies the effects of innate immunity on cystitis is complex and not fully determined. It is known that the phenotypic adaptations of macrophages during the interaction are dynamic and involve not only changes in major functionality (M1 versus M2), but also some delicate changes in certain function such as phagocytotic, regenerative or immune resolution (22).

Here in this study, we found that diabetes likely affected the phenotype of macrophages in such a delicate manner, since diabetes seemed to alter the levels of markers for macrophage functionality, iNOS, TNFα and IFNγ, and the levels of PCNA, a cell proliferation marker (23–25). Interestingly, the effects of diabetes on cystitis are at least partially contributable to the increases in NLRP3, a regulator for IL-1β. It was supported by our *in vivo* experiment clearly showing a loss of aggravated cystitis in diabetes by macrophage-depletion of NLRP3.

The NLRP3 inflammasome has been found activated by many different stimuli, such as the increased extracellular glucose level in a diabetic setting (26). However, the exact molecular regulation of NLRP3 activation appeared to be complex and controlled by multiple factors (27, 28). It has been reported that NLRP3 is activated in different types of diabetes, while NLRP3 depletion seemed to have a protective effect against diabetes (29, 30).

Based on our findings in the current study, the increased proliferation, migration and the phagocytosis of bladder macrophages in a diabetic status appeared to be harmful to individuals baring cystitis (31, 32). It is believed that exaggerated inflammation could have an adverse effect on the normal immune reaction and its resolution (33). Apparently, diabetes can be such a condition that causes hyperreaction of the immune cells and the worsening of the disease.

To summarize, our data suggest that macrophage-depletion of NLRP3 attenuates the severity of cystitis in a diabetic status. Further exploration of the molecular mechanisms may improve our knowledge on the pathological and molecular events happening during cystitis to allow development of novel therapies.

## Data availability statement

The original contributions presented in the study are included in the article/supplementary material. Further inquiries can be directed to the corresponding author.

## Ethics statement

The animal study was reviewed and approved by Shanghai Jiao Tong University School of Medicine.

## Author contributions

YP and YG are responsible for data acquisition and analysis. YP are responsible for study conception and design, data acquisition and analysis. YP wrote the manuscript and all authors have read the manuscript and agreed with the publication. YP are responsible for funding and are the guarantee of the study. All authors contributed to the article and approved the submitted version.

## Funding

This work was supported by internal funding from RenJi Hospital.

## Conflict of interest

The authors declare that the research was conducted in the absence of any commercial or financial relationships that could be construed as a potential conflict of interest.

## Publisher’s note

All claims expressed in this article are solely those of the authors and do not necessarily represent those of their affiliated organizations, or those of the publisher, the editors and the reviewers. Any product that may be evaluated in this article, or claim that may be made by its manufacturer, is not guaranteed or endorsed by the publisher.
